# Effect of l-theanine tablets in reducing stress-related emotional signs in cats: an open-label field study

**DOI:** 10.1186/s13620-018-0130-4

**Published:** 2018-10-09

**Authors:** V Dramard, L Kern, J Hofmans, C A Rème, C S Nicolas, V Chala, C Navarro

**Affiliations:** 1 Referral Behaviour Veterinary Clinic, 16 rue Jeanne d’Arc, 69003 Lyon, France; 2 Referral Behaviour Veterinary Clinic, 6 place Léon Deubel, 75016 Paris, France; 3 Veterinary Clinic, Avenue des Martyrs 173, 4620 Fléron, Belgium; 4 Medical Department, Virbac, 13ème rue, LID, 06511 Carros, France

**Keywords:** L-theanine, Undesirable behaviours, Stress, Anxitane®, Cats, Field

## Abstract

**Background:**

L-theanine is an aminoacid found in tea leaves which has relaxing effects in humans and animals. It is a structural analogue of glutamate which can bind glutamate receptors. Although the relaxing action of L-theanine has been shown in humans, laboratory animals and dogs, it has never been published in cats. The goal of this open-label, multicentre and prospective trial was to determine whether an L-theanine based oral supplement (Anxitane®, Virbac, France) could attenuate manifestations of stress in cats under field conditions.

**Case presentation:**

Thirty-three privately owned cats presenting signs associated with stress or fear (inappropriate urination/defecation, fear-induced aggressiveness, hypervigilance/tenseness or physical/functional manifestations of stress) for at least 1 month, were included in the study. They were given L-theanine (Anxitane®, 25 mg twice a day) for 30 days and 20 stress-related parameters were scored at Days 0, 15 and 30. The evolution of some parameters was also rated relative to Day 0. All median scores of the 20 parameters were significantly reduced at D30, and 30/33 cats (91%) had a reduced global score at the end of the study, including 21/33 with ≥50% score reduction. The median (IQR) global scores went from 18 (13–23) at D0 to 11 (8–13) at D15 and 5 (3–12) at D30 (*p* < 0.0001; Friedman test; significant reduction starting from D15). All the stress-related signs were significantly improved compared to D0, according to the owners, especially inappropriate elimination. Tablet palatability was judged good or very good in 94% of cases with spontaneous intake by cats when given by hand or in food. Tolerance was satisfactory as well, and no side effects were reported, so that most owners (27/33; 82%) were satisfied with the product.

**Conclusions:**

Despite the lack of a placebo group, it can be concluded that L-theanine (Anxitane®) helped to improve the undesirable manifestations of stress in cats in as soon as 15 days, though better results could be seen after 30 days of administration. These encouraging results show that L-theanine can help manage stress-related behaviour, but additional trials with a placebo group should be run to confirm this effect.

## Background

Green tea has been used worldwide for centuries and is very much appreciated for its taste and relaxing properties [1, 2]. After the discovery that theanine, an amino acid, was the main component of green tea leaves *(Camellia sinensis)* responsible for the relaxing effect, many studies have investigated the properties of this molecule (see [2–4] for reviews). L-theanine is the main bioavailable form present in tea (1–2% of total leaf weight), and represents around 50% of total free amino acids in tea [2]. In mammals, L-theanine can cross the blood brain barrier, in a dose-dependent manner, in the first hour after administration and remains in the plasma and brain for a few hours [2, 5, 6]. It can now be synthesised or extracted from tea and can be used as an oral dietary supplement for humans or animals.

In humans, L-theanine has been shown to facilitate the generation of alpha waves in the brain that are indicative of a relaxed, awake and alert state, without promoting drowsiness [2, 7–9]. L-theanine would also help regulate the physiological parameters usually increased during stressful events (e.g. blood pressure, heart rate, cortisol secretion), which further contributes to the stress-reducing effects [9–12]. L-theanine’s relaxing effect has also been shown in dogs with fear of humans [13] or storms [14]. After 1 or 2 months of L-theanine administration (twice a day), fearful reactions were reduced. Finally, L-theanine would enable the increase of learning capacities [15, 16].

The relaxing properties of L-theanine (or γ-ethylamino-L-glutamic acid) can mainly be explained by its similarity in structure to glutamate, the most important excitatory neurotransmitter in the brain, which allows the compound to interact with glutamate receptors such as AMPA, kainate, and NMDA (at the glycine site). This antagonistic activity may play a role in neuroprotection against glutamate toxicity [4, 9, 17], and in secretion of dopamine and serotonin in specific brain areas [18].One study also reported the increase of GABA concentration in mice brain following L-theanine administration: this effect probably explains the counteracting activity of L-theanine against caffeine [17]. These effects likely explain the ability of L-theanine to modulate neuronal response involved in mood, stress, pleasure and reward [4, 17–21].

Although the relaxing activity of L-theanine has been shown in humans, laboratory animals and dogs, it has not been published in cats. Cats can show undesirable behaviours when experiencing stressful events, such as urinary marking of furnitures and household structures, excessive scratching, aggressiveness and overgrooming. In addition to these behavioural changes, stress can also lead to recurring physical conditions such as emotional (often qualified as idiopathic) cystitis, chronic digestive signs (vomiting, diarrhoea) and disturbed eating behaviours [22–24]. From the pet owner’s stand point, the most unwanted behaviours are urinary marking [25, 26] and excessive scratching [27], which are usually the first signs of discomfort. Before the situation worsens and requires medication, it would be useful to determine if an oral supplement containing L-theanine can reduce stress-related behaviours as in the other species studied so far.

The aim of the present study was to evaluate whether a veterinary L-theanine-based oral supplement (Anxitane® tablets, Virbac) could attenuate signs of stress in cats under field conditions.

## Materials and methods

### Aim, design and settings

This study was an open-label, multicentre and prospective trial designed to evaluate the impact of L-theanine (Anxitane®) on stress or fear-related reactions, in client-owned cats, in the field. The cats were included in the study regardless of age, breed, sex, origin (conditions of adoption) or lifestyle (indoor or outdoor cats). Of the 11 investigators, 9 were located in France (from 5 regions) and 2 were in Belgium (from 2 different areas). Amoung the 11 investigators 5 of them hold the French Inter School Diploma of Behaviorist Veterinarian and exerted in referral practice.

## Case presentation

Inclusion criteria were: occurrence, for at least 1 month, of one or more of the following manifestations of stress: inappropriate urination / defecation, aggressiveness (from fear), hypervigilance / tenseness, inhibition, cohabitation problems, fear of humans or physical manifestations of stress (digestive signs, hypersalivation, excessive body licking or changes in feeding or drinking behaviour).

All cats had to be in good health for inclusion in the study, on the basis of history and a complete physical examination. Clinic pathological testing was not considered mandatory for inclusion so as to avoid stress conditions or animal sedation. Exclusion criteria were: cats with serious chronic illness, or cats receiving supplement products, pheromones or having received any other psychotropic medications for the behaviour condition in the preceding week.

### Product and administration

The cats received 25 mg (half a tablet of Anxitane® S, Virbac, France) in the morning and 25 mg in the evening, corresponding to 50 mg of L-theanine per day, for 1 month. Any other oral supplement was forbidden, as well as pheromone applications or drugs. No specific behavioural therapy instructions were given, so as to keep a reproducible environment among the subjects. Nevertheless standard recommendations regarding the feline species were given to each owner (no punishment, elementary rules for feeding, etc).

All cats were treated against external parasites (fipronil spot-on) and internal parasites (milbemycine/praziquantel) at recommended dosages prior to the inclusion.

### Interventions and assessments

Each cat was assessed on Days 0, 15 and 30 (D0, D15 and D30), with a physical examination on D0 and D30.

A behavioural examination based on 20 parameters - rated from 0 to 3 - was performed by the veterinarian, according to the owner’s statement. Those parameters included:

- Neurovegetative signs (9 parameters): polypnea; shivering; mydriasis; piloerection; rolling skin syndrome; vomiting; diarrhoea; hypersalivation; fear-induced urination or defecation;
- Interspecific interactions (6 parameters): meowing; excessive demands for attention; threats (hissing) or even bites because of irritation (when patted or while playing with the owner); threats (hissing) or even attacks when surprised or because of fear; aggression redirected against people / objects / furniture / tail; avoidance behaviour;
- Emotional state and its postural expression (5 parameters): inhibition (flattened ears / tendency to crouch/hide); agitation; hypervigilance and hyperaesthesia; tendency to panic / run away or hide; inhibition with licking / stereotypical behaviour.

The scores for the 20 parameters were added to calculate a global score reflecting the presence of undesirable behaviours possibly related to stress in test animals.

The investigators and the owners also had to rate, on a 0% (complete improvement) to 100% (no improvement) scale, their perception of the evolution in the 4 dominant signs as compared to baseline evaluation; these signs were considered stress manifestations: inappropriate elimination; aggressiveness; stress-related signs (hypervigilance, nervousness, fear) and stress-induced functional/organic signs (digestive signs, disturbed eating and drinking behaviours, overgrooming and scratching).

Four other parameters describing elementary behaviours (mandatory for animal survival) were scored, from 0 (normal/regular) to 2 (very disturbed) according to their intensity or frequency: sleep (normal to agitated), appetite (normal/regular to disturbed), drinking (normal/regular to high water consumption) and mood (happy to sad).

Parameters used for behaviour assessment rely on common consensus among French and Belgian behaviourist associations. These recommendations are based upon several publications [28–31].

The product itself was then evaluated for acceptability, tolerance and owner satisfaction. The first two parameters were scored from 1 (very good) to 4 (poor).

### Statistical analysis

The evolution of the parameters over time was compared using the Friedman test followed by adjusted (Holm) pairwise comparisons using the Wilcoxon signed rank test, in case of significance. Only statistical tests giving *p* < 0.05 were considered significant. Data are presented as median (range) or median (interquartile range = IQR).

## Results

In total, 33 cats aged 5 months to 16 years and weighing between 2 and 10.1 kg were included in the study (Table 1). None were excluded after the start of the study. Most cats were European shorthair cats, had been adopted before the age of 1 year and generally came from a relative or a friend, or had been found (Table 1).

**Table 1:** Demographic data and reason for inclusion of the 33 cats

Breed *n* (%)	European	28 (85%)
	Persian	3 (9%)
	Siamese	1(3%)
	Somali	1 (3%)
Age (year)	median (range)	4,3 (0.4–16)
Weight (kg)	median (range)	4,5 (2–10)
Age of adoption (year)	median (range)	0,2 (0–12)
Origin *n* (%)	Friend or relative	11 (33%)
	Found	11 (33%)
	Breeder	3 (9%)
	Shelter	3 (9%)
	Pet shop	1 (3%)
	Other	4 (12%)
Main reason for inclusion *n* (%) (an individual cat maypresent several signs)	Stress-related signs	26 (79%)
	inappropriate elimination	15 (45%)
	aggressiveness	8 (24%)
	stress-induced functional/organic signs	7 (21%)
Age of onset (year)	median (range)	1 (0.2–12)
Duration of signs (year)	median (range)	1.8 (0.08–10)

The main reasons for inclusion were stress-related behavioural signs (hypervigilance, nervousness, fear) in 79% of the cases and inappropriate elimination (45% of the cases) while aggressiveness (24% of the cases) and stress-induced functional/organic signs (mainly changes in feeding or drinking behaviour; 21% of the cases) occurred less often (Table 1). The median (range) age of onset of undesirable behaviours/signs was 1 (0.2–12) year with 67% and 15% of the cats starting to show issues before the age of 1 and from adoption, respectively. In 42% of the cats, such behaviours had been stable since their onset, but had worsened over time in another 58% of the cats.

### Scores of stress-related parameters

All the parameters significantly improved (lower score) during the course of the study, except for those which concerned too few animals (< 4 cats: diarrhoea, hypersalivation and redirected aggression). Hypervigilance/hyperesthesia was the parameter with the highest median score at D0 and concerned 27/33 cats (82%). A significant decrease of this median (IQR) score was observed during the course of the study: from 3 (2–3), to 1 (0–2) and 1 (0–1) at D0, D15 and D30 respectively (*p* < 0.0001). At the end of the study, 8/27 cats (30%) no longer showed the signs. Shivering was the parameter most reduced in frequency since it was detected in 11/33 cats (33%) at the beginning of the trial and in only 3/33 cats (9%) by the end of the study.

**Fig. 1:**
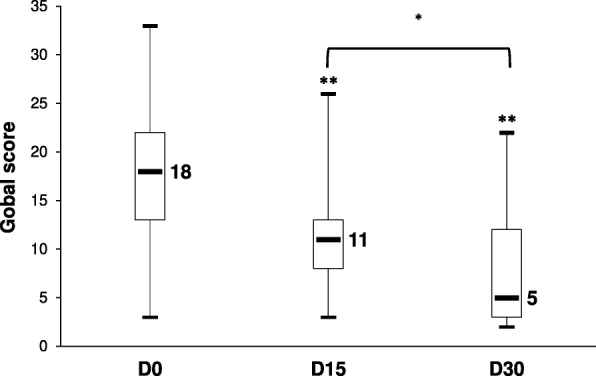
Evolution of the global score (sum of the scores for the 20 stress-related parameters evaluated) over time during the study. Data are presented as median/1st and 3d quartiles/minimum and maximum values. ** *p* < 0.01 compared to D0 according to adjusted pairwise comparison (Friedman test: *p* < 0.0001)

**Fig. 2:**
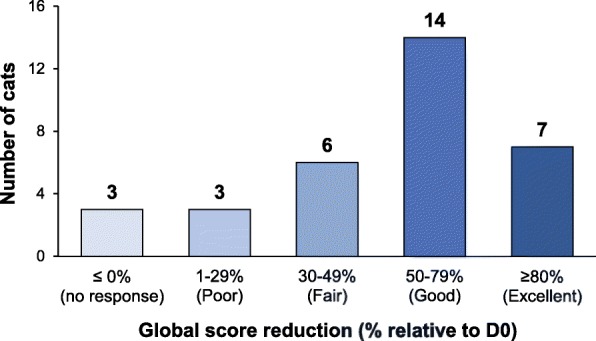
Distribution of the cats according to their response to L-theanine. The reduction in the global score reflecting stress-related parameters was calculated at D30 relative to D0 (%) for each cat. The number of cats showing no reduction (no response to L-theanine), a 1–29% reduction (poor response), a 30–49% reduction (fair response), a 50–79% reduction (good response) or a reduction of 80% or more (excellent response to L-theanine) in their global score, are reported (total *n* = 33)

The median (IQR) global score significantly decreased from D0: 18 (13–23) to D15: 11 (8–13) and D30: 5 (3–12); *p* < 0.0001 (Friedman test); Fig. 1. Improvement between D15 and D30 was also statistically significant; (*p* < 0.05); Fig. 1.The median (IQR) relative reduction of the global score from D0 to D30 was 62% (39–75%). Most of the cats (30/33; 91%) had a reduced score at the end of L-theanine administration and only 3/33 cats (9%) showed no improvement (Fig. 2). The global score was reduced by 50% or more in most cats (21/33; 82%), with 7/33 (21%) showing an excellent response to L-theanine (score reduced by 80% or more; Fig. 2).

### Improvement of signs (owner perception)

According to the owners, among the 4 stress-related signs which were evaluated, the inappropriate elimination (urination/defecation) was the one that improved the most (Table 2). At the end of the study, 10/18 cats (66%) were deemed to have stopped showing this inappropriate behaviour (score of 0% at D30) and 5/13 (38%) to have stopped showing signs of disturbed grooming, disturbed food or water intake, or digestive signs. Hypervigilance/tenseness/fear and aggressiveness were the least improved parameters, with 3/31 (10%) and 3/16 (19%) cats, respectively, deemed to have stopped showing those signs. Still, all the parameters significantly improved according to the median of ratios given by the owners (Table 2).

**Table 2:** Relative severity of stress-related signs, compared to D0 (%)

	D0 (default value)	D15	D30	Friedman *p*-value
Inappropriate urination/defecation (*n* = 18)	100	23 (0–90)*	0 (0–44)**	< 0.001
Stress-induced functional/organic signs (*n* = 13)	100	28 (15–55)**	20 (0–25)**	< 0.001
Hypervigilance/tenseness/fear (*n* = 31)	100	60 (50–79)**	25 (18–50)**	< 0.001
Aggressiveness (*n* = 16)	100	80 (38–100)*	40 (13–65)**	< 0.001

A default value of 100% was set at D0 and the severity of signs was rated afterwards relative to this value, for each cat. A value of 0% meant a total improvement while a value of 100% meant no improvement. All values for each parameters ranged from 0 to 100 at any day. The values reported here are the median (IQR). *p < 0.05 and **p < 0.01 compared to Day 0 according to adjusted pairwise comparisons

### Sleep, appetite, drinking and mood evaluation

Of the 12 cats with sleeping issues at D0 (score > 0), 9/12 (75%) were judged normal at the end of the study (significant reduction of scores, Friedman test, *p* < 0.05). Similarly, of the 16 cats described as sad or unhappy (score > 0 for mood), 12/16 (75%) were normal/happy at the end of L-theanine administration (significant reduction in scores, Friedman test, *p* < 0.001). Appetite and drinking behaviours also improved in some cats but not significantly. 2/9 (22%) and 1/2 (50%) of the cats with appetite or drinking issues at baseline became normal (score of 0) at the end of the study.

### Ease of administration, tolerance and owner’s satisfaction

**Fig. 3:**
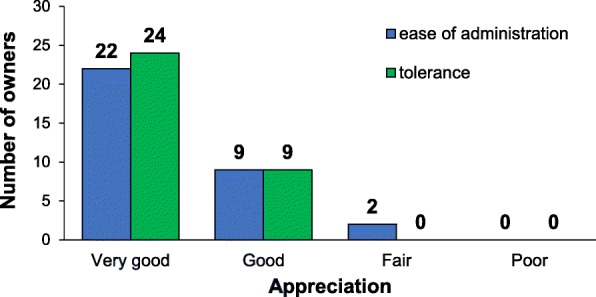
Ease of administration and tolerance of Anxitane®. The owners had to judge the ease of administration of the tablets (blue) and their tolerance (green). The number of owners judging these parameters as very good, good, fair or poor are reported here

The ease of administration of the tablets was considered to be very good by 22/33 (67%) owners, good by 9/33 (27%) and fair by 2/33 (6%) (Fig. 3). The median (IQR) score for this parameter was of 1 (1–2) on a 1–4 scale. Tolerance was also deemed very good or good by all the owners (Fig. 3) with the same median (IQR) score. Most owners were very satisfied or satisfied [13/33 (39.5%) and 14/33 (42.5%), respectively] and only 6/33 (18%) owners were poorly or not satisfied (Fig. 3).

### Adverse events

No serious adverse events were reported during the course of the study. Two cats were more sleepy/apathetic during the first days of administration, leading to a reduction of doses (once a day instead of twice a day) in one of them. Both cats improved (following the dose reduction for the first one and spontaneously within 3 days for the second one) and were kept in the trial.

## Discussion

Almost all of the parameters evaluated were improved during the course of this study in 33 cats with stress-related signs. The behaviour of most of the cats improved after 15 or 30 days of L-theanine administration, leading to high owner satisfaction.

However, this study was open-label and did not contain a control group. Conclusions should therefore be taken with caution as owners/vets could have expected some sort of improvement during this type of trial. Still, the extent of the decrease in undesirable stress-related signs observed during the study in most cats is promising and suggests that L-theanine could calm and relax cats. Especially, some signs like inappropriate urinating, excessive grooming, hypervigilance, excessive demands of attention or panicking were improved as of Day 15. Irritability also decreased and cats were found to be more calm and gentle at the end of the study.

Among stress-related signs, inappropriate urination (whether due to excessive marking or inappropriate elimination) was probably the most bothersome for the owners and is one of the most frequent causes of consultation [24–27]. This type of sign is mainly due to a lack of adaptation to the environment or to the social conflicts a cat faces on a daily basis. Interestingly, inappropriate urination disappeared in 66% of the cats in this study, despite the lack of a specific behavioural therapy.

In cats (and dogs), many factors can induce stress-related behavioural signs [23–25]: an unpleasant experience during development, unsuitable living conditions and environment, bad relationships with owners or other animals, or pain and pathological conditions. Conflicts with the owners can create and help perpetuate unwanted signs, and the reverse can be true as well. Behavioural therapy can help owners understand and face the condition and adapt their behaviour or modify the cat’s environment in order to reduce feline stress. Instead of reprimanding the animal (especially in case of inappropriate urination or nervousness), the owner will be more understanding which in turn will help the cat. Although behavioural therapy is the mainstay of management of behavioural issues, pheromones, nutritional supplements or psychoactive drugs can also be prescribed to decrease anxiety [22]. In the current study, although no specific behavioural modifications were implemented, some basic behavioural therapy advice was given to the owners. It is possible that this advice contributed, at least in part, to the results obtained. We feel, however, that the important reduction in unwanted signs recorded in this study cannot be attributed solely to owners given the absence of specific behavioural advice provided by veterinarians. Further studies could be performed to assess potential additional or synergistic benefits of Anxitane® tablets over behavioural therapy alone.

Over time, it was found in this trial that most signs significantly improved as of Day 15. Since product tolerance was very good, a longer administration over 2–3 months could have helped reduce further unwanted signs still detected at 1 month in some cats, like aggressiveness or hypervigilance/tenseness/fear. Indeed, it is generally advised to carry on administration of a stress reducing product for a few months since effects can be seen only after 8 or 16 weeks of administration [13, 32].

In this study, L-theanine significantly helped improve the sleep and mood of cats but did not have the same impact on disturbed eating and drinking behaviours. Again, a longer administration could have helped reduce the issues but other concurrent ailments (endocrine or renal, for example) cannot be excluded and should be checked in case the behavioural signs persist.

Although no study describing the mechanism of action of L-theanine in cat is available, it is highly likely that it is similar to what was described earlier in laboratory animals (especially, an action on glutamate receptors and release of neuromediators) [4, 17, 19, 21]. More specifically, no sedative effect of L theanine has been previously reported in human, in rats, nor in dogs [2, 7, 8, 13, 14] .Therefore we expect that the effects observed in this study are due to a decrease of the stress level (and not to a sedative effect).

Thus, as in dogs [13, 14], L-theanine can be of value as an oral dietary supplement in cats, as it is safe and easy to give to individuals presenting with signs of stress (hypervigilance, mydriasis, rolling skin syndrome, excessive meowing, inappropriate elimination, etc.), in order to help them cope with changes in their environment.

## Conclusion

Cats are sensitive to environmental changes (e.g. change in territory or owners and occasional stressful events) and can show discrepancies with the feline ethogram. Reactions can vary among cats and some may develop behavioural signs or even functional/organic signs. They can become hypervigilant, show signs of fear, and in many cases, urinate inappropriately. These behaviours are unwanted for both the cats and their owners, and the latter can increase feline stress by the way they handle the situation. Although this study does not contain any control group, it suggests that in stressed cats, administration of L-theanine (Anxitane®) twice daily may help improve undesirable signs in as soon as 15 days, though better results were seen after 30 days of administration. These encouraging results showed that L-theanine can help manage stress related behaviour, although additional trials with a placebo group should be run to confirm this effect.
